# Brentuximab Vedotin in Hodgkin Lymphoma and Anaplastic Large Cell Lymphoma

**DOI:** 10.6004/jadpro.2012.3.3.8

**Published:** 2012-05-01

**Authors:** Vivian Tsang

**Affiliations:** From Cedars-Sinai Medical Center, Los Angeles, California

Lymphoma, a condition that is characterized by an abnormal growth of lymphocytes, can be classified into two main categories: Hodgkin lymphoma (HL) and non-Hodgkin lymphomas (NHL). Anaplastic large cell lymphoma (ALCL) is a subset of peripheral T-cell NHL.

In general, HL responds well to conventional chemotherapy such as ABVD (doxorubicin [Adriamycin], bleomycin, vinblastine, dacarbazine). Likewise, anaplastic lymphoma kinase-1 (ALK1)-positive ALCL responds well to standard CHOP (cyclophosphamide, doxorubicin, vincristine [Oncovin], and prednisone) therapy as compared with the ALK1-negative subset. The ALK gene is found on chromosome 2 and has the ability to form fusion proteins. When ALK protein overexpression occurs, antibody to the protein is developed (Savage et al., 2008). ALK protein expression is considered to be an independent prognostic factor in predicting clinical outcomes.

The ALK-positive subset typically occurs in younger patients and has a significantly better prognosis than does the ALK-negative subset (Barreca et al., 2011; Gascoyne et al., 1999). But despite a high rate of cure following front-line chemotherapeutic agents, the incidence of refractory or resistant disease still persists, even after autologous hematopoietic stem cell transplantation. After exhausting several lines of therapy, patients with refractory disease may run out of options. However, brentuximab vedotin (Adcetris), a targeted anti-CD30 (cluster of differentiation 30) monoclonal antibody-drug conjugate (ADC), was recently introduced for the treatment of HL and ALCL (Younes, 2009; Lymphoma Research Foundation, 2012).

## Drug Design

Brentuximab vedotin, also known as SGN-35, is uniquely designed as a dual-action agent. The initial development of this drug followed a path similar to that of many monoclonal antibodies with specific targets. CD30 is an antigen that is commonly expressed in selected lymphoid and nonlymphoid malignancies, particularly in the Reed-Sternberg cells of Hodgkin lymphoma and anaplastic large cell lymphoma. For this reason, the initial design plan was to develop an anti-CD30 antibody. Despite the intended mechanism of action for targeting CD30, the initial results of the monoclonal antibody were disappointing (Ansell et al., 2007; Bartlett et al., 2008). To enhance the antitumor effects, a tubulin inhibitor was added to the monoclonal antibody.

The ADC was designed by linking a chimeric anti-CD30 monoclonal antibody with a cytotoxic synthetic tubulin polymerization inhibitor monomethyl auristatin E (MMAE) via the valine-citrulline peptide (Okeley et al., 2010). After the complex is formed and internalized into the target cells, lysosomal enzymes cleave and release the cytotoxic MMAE, resulting in the inhibition of microtubule formation. This selective delivery approach not only enhances a drug’s activity by enabling it to reach a specific target site, but also minimizes potential toxic adverse events (Okeley et al., 2010; Deutsch, Tadmor, Podack, & Rosenblagg, 2011).

Brentuximab vedotin exerts its activity in the G2/M phase, resulting in cell cycle arrest and apoptosis of CD30-positive tumor cells (Younes et al., 2010). Historically, there has only been one other agent with a similar ADC design: an anti-CD33 monoclonal antibody known as gemtuzumab ozogamicin, which was voluntarily withdrawn from the market in June 2010 due to a lack of survival benefit.

## Dose-Finding Study

Brentuximab vedotin was studied in a phase I, open-label, multicenter dose-escalation trial in 45 patients with CD30-positive hematologic malignancies (Younes et al., 2010). Intravenous brentuximab vedotin was administered at 0.1–3.6 mg/kg every 3 weeks and evaluated every 6 weeks. This study included a traditional dose-escalation method followed by a cohort-expansion phase. The eligibility criteria for this study included patients who were at least 18 years of age, with tumor measuring at least 10 mm in diameter and an Eastern Cooperative Oncology Group performance status of 2 or lower (based on a scale of 0 to 5, with 5 representing the most debilitated state). Patients were excluded if they had a prior allogeneic stem cell transplant. Baseline characteristics are presented in Table 1.

**Table 1:**
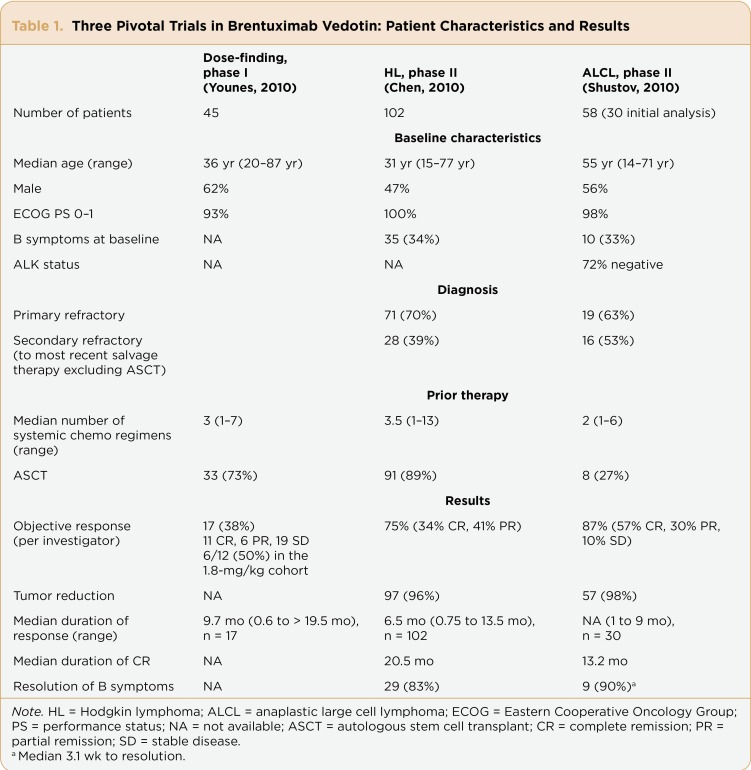
Table 1. Three Pivotal Trials in Brentuximab Vedotin: Patient Characteristics and Results

Of the 45 patients, 42 had HL, 2 had ALCL, and 1 had angioimmunoblastic T-cell lymphoma. Twenty-one patients (47%) had stage II disease; the remaining patients had stages III and IV disease (approximately 25% for each stage). Patients were taken off study for disease progression, but observation continued for an additional 30 days after the last brentuximab vedotin dose or after a new treatment was initiated. The primary objectives were to define the safety profile and to determine the maximum tolerated dose (MTD) of brentuximab vedotin. The secondary objectives were to determine the pharmacokinetics measures for ADC and MMAE, evaluate immunogenicity, and assess antitumor response (Younes et al., 2010).

Three patients (25%) in the 2.7-mg/kg cohort developed unacceptable dose-limiting toxic effects, which included unrelated acute renal failure, hyperglycemia, unrelated prostatitis, and febrile neutropenia, all evaluated as grade 3 toxicities. As a result, 1.8 mg/kg was considered the MTD, or the highest dose that did not result in unacceptable toxic effects. The most common adverse events included fatigue, pyrexia, diarrhea, nausea, neutropenia, and peripheral neuropathy. Serious adverse events thought to be related to brentuximab vedotin included grade 3 hypercalcemia, myocardial ischemia, anaphylactic reaction, peripheral sensorimotor neuropathy, pyrexia, and abdominal pain. Similar to other antitubulin or antimicrotubule agents, peripheral neuropathy is considered a class effect (Swain & Arezzo, 2008).Although most cases of peripheral neuropathy presented in this study were grade 1 or 2 sensory findings, these symptoms were considered clinically significant. The one patient in the 3.6-mg/kg cohort experienced febrile neutropenia complicated with presumed sepsis, which ultimately resulted in unexpected mortality (grade 5 toxicity) 14 days after receiving the initial dose (Younes et al., 2010).

In terms of pharmacokinetic and pharmacodynamic parameters, the median time to maximum concentration (C_max_) following infusion was almost immediate for the ADC and approximately 2 to 3 days for MMAE. The estimated half-lives for the ADC and MMAE were 4 to 6 days and 3 to 4 days, respectively. The steady states for both the ADC and MMAE occurred by approximately 21 days, as illustrated by the concentration-time curves (Younes et al., 2010).

Of the 45 patients studied, 17 patients had notable objective response (11 complete remissions and 6 partial remissions) and 19 had stable disease post brentuximab vedotin ADC. Objective response was observed in 6 of 12 patients (50%) in the 1.8-mg/kg cohort, which was the MTD cohort. Both ALCL patients in the study reached a complete remission. The median duration of response for the 17 patients with notable objective response was 9.7 months (range, 0.6 to > 19.5 months). The median progression-free survival was 5.9 months. In addition, a trend for extended progression-free survival was toward if the dose received was at least 1.2 mg/kg (Younes et al., 2010).

The dose-finding study concluded that administration of brentuximab vedotin produced a lasting objective response while promoting tumor regression in patients with CD30-positive relapsed or refractory HL and ALCL. With the determination of the MTD, most adverse events were relatively low grade and could be managed with routine supportive care.

## Pivotal Phase II Trials

The first of two pivotal trials was a phase II, single-arm, multicenter study evaluating the efficacy and safety of brentuximab vedotin in 102 patients with relapsed or refractory HL post–autologous stem cell transplant (ASCT; Chen et al., 2010). The second pivotal trial was a phase II, single-arm, multicenter study evaluating the efficacy and safety of brentuximab vedotin in 58 patients with relapsed or refractory systemic ALCL (Shustov et al., 2010). The eligibility criteria for both of these studies were similar in that they included patients with CD30-positive disease proven by histology, measurable disease of at least 1.5 cm by computed tomography or positron emission tomography (PET), who were >= 12 years old (or >= 18 years old for the international sites). In post-ASCT patients, the time interval between the start of the study and prior autologous transplant needed to be at least 12 weeks. For the ALCL population, documentation of ALK status was required. In both trials, brentuximab vedotin 1.8 mg/kg was administered as a 30-minute intravenous infusion every 3 weeks for up to 16 cycles. The primary endpoint for both studies was overall objective response (Chen et al., 2010; Shustov et al., 2010).

In the Chen study, 102 patients with relapsed or refractory HL post-ASCT were enrolled into the study across 26 centers. Thirty-five (34%) had B symptoms at the initiation of the study. The objective response rate was 75% (34% complete remission [CR]; 41% partial remission [PR]). Tumor reduction was observed in 97 patients (96%), which was comparable to the findings from the phase I trial. Patients were on treatment for a median duration of 27 weeks (range, 3–54 weeks) and received a median of 9 cycles (range, 1–16 cycles) of brentuximab vedotin. Of the patients who initially presented with B symptoms, 29 patients (83%) had symptom resolution by a median of 3 weeks (range, < 1–16 weeks; Chen et al., 2010).

In the Shustov study, 58 patients with relapsed or refractory ALCL were enrolled. An interim analysis was conducted after the first 30 patients were enrolled. Ten patients (33%) had B symptoms at the initiation of the study. The overall response rate was 87% (57% CR, 30% PR, 10% stable disease, 3% not able to assess). Tumor shrinkage occurred in all but one patient. The presence or absence of ALK1 did not differ among those who reached a CR or PR. Patients had a durable response from 4 to 36 weeks, and median time to objective response was obtained in 6 weeks (range, 5–12 weeks). Of the patients who initially presented with B symptoms, nine (90%) had symptom resolution (Shustov et al., 2010).

See Table 1 for a summary of these three pivotal trials, including baseline characteristics and results.

## Adverse Events

Based on the findings of the two pivotal studies, the most commonly observed adverse events of any grade included peripheral sensory neuropathy, fatigue, nausea, neutropenia, diarrhea, dyspnea, insomnia, and pyrexia. Grades 3 and 4 adverse events included neutropenia, peripheral sensory neuropathy, thrombocytopenia, diarrhea, anemia, hyperglycemia, abdominal pain, and pulmonary embolism. There were no grade 5 adverse events observed in either trial. The specific incidence of each of the above-mentioned adverse events is presented in Table 2.

**Table 2:**
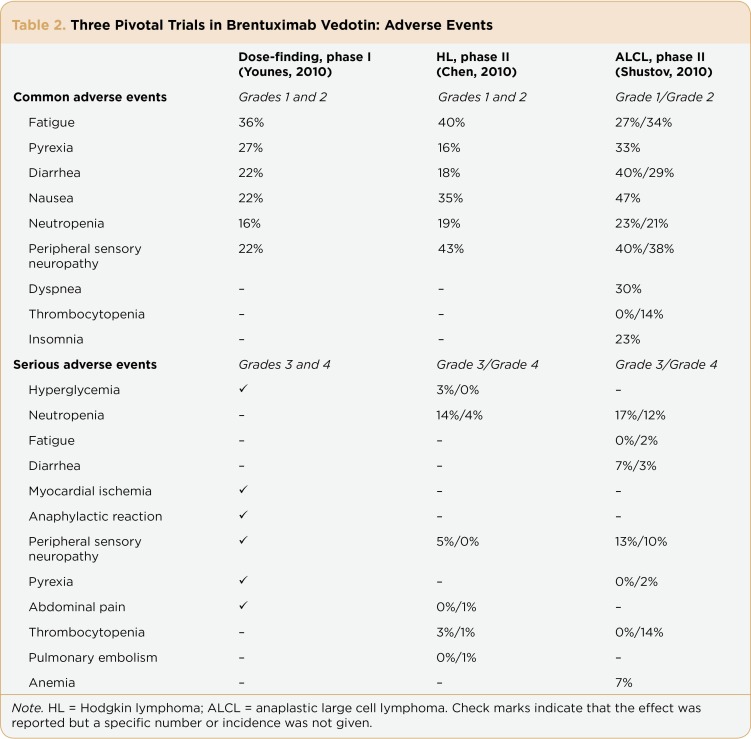
Table 2. Three Pivotal Trials in Brentuximab Vedotin: Adverse Events

## FDA Approval

Brentuximab vedotin induced noticeable tumor shrinkage, resolution of B symptoms, and manageable adverse events in both of the key phase II studies discussed in this article. In light of these favorable outcomes, the US Food and Drug Administration (FDA) approved the use of brentuximab vedotin for dual indications, including HL and ALCL (Chen et al., 2010; Shustov et al., 2010).

Since the approval of brentuximab vedotin in August 2011, the FDA has issued a new boxed warning detailing the incidence of progressive multifocal leukoencephalopathy; potential signs and symptoms may include behavioral or mood changes, visual changes, impaired speech, and decreased strength, which may develop over the course of weeks to months. In addition, brentuximab vedotin is now contraindicated with the use of bleomycin, as it may potentiate the risk of pulmonary toxicity (FDA, 2012).

## Implications

Hodgkin lymphoma and ALCL are two rare hematologic conditions that are both treated with chemotherapy. Despite the fact that they may have some response to standard treatments, a small proportion of patients have disease that is resistant to durable response. Autologous stem cell transplantation is one treatment option, but it is not suitable for every patient. Agents such as rituximab (Rituxan) and pralatrexate (Folotyn) may play a role in treatment, but the overall response to these agents is limited and temporary.

Brentuximab vedotin has undergone numerous clinical trials in the evaluation of safety and efficacy in CD30-positive hematologic malignancies. With the data provided by the two pivotal trials, brentuximab vedotin demonstrated an improved objective response for both HL and ALCL. In addition, the dose-finding trial demonstrated brentuximab vedotin to be safe when administered at the MTD. Peripheral neuropathy, the most common adverse event, is generally self-resolving after discontinuation of therapy. With its specific design as an ADC, brentuximab vedotin shows enhanced activity while minimizing toxicity. Currently, brentuximab vedotin has been approved for the treatment of patients with Hodgkin lymphoma after relapsing post-ASCT, or those who have failed to respond to at least two prior regimens and are not transplant candidates, as well as patients with anaplastic large cell lymphoma who remain refractory after at least one prior regimen.

Based on the current evidence, advanced practitioners could offer brentuximab vedotin to patients with CD30-positive HL or ALCL, as it may offer the potential for long-term remission, particularly in those with refractory disease or post–ASCT. See Table 3 for a summary of key information on this agent.

**Table 3:**
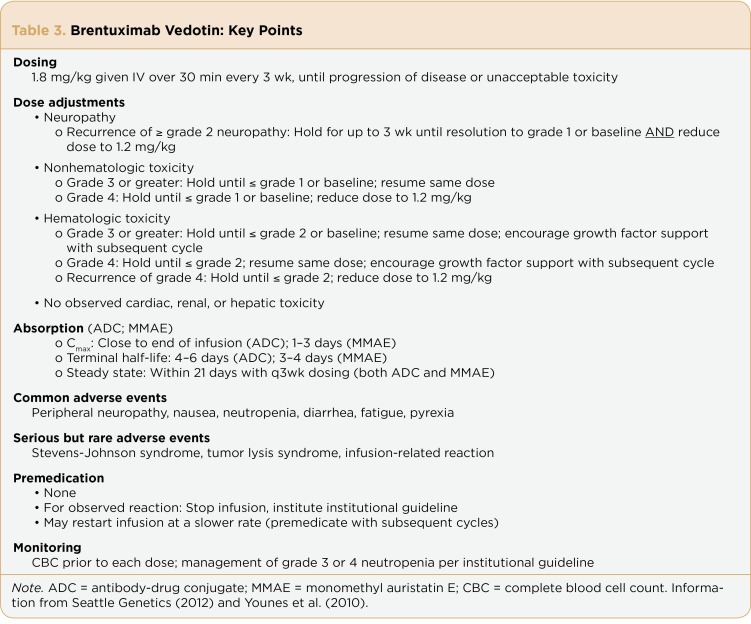
Table 3. Brentuximab Vedotin: Key Points

In addition, brentuximab vedotin is also being evaluated as an up-front treatment option in CD30-positive hematologic malignancies regardless of transplant status, in the pediatric population, and in nonlymphomatous malignancies either as a single agent or in combination with another regimen. Brentuximab vedotin could potentially gain expanded indications with the results of upcoming clinical trials data (National Institutes of Health, 2012).
